# Study on Differences in Lipid Composition of Camel Milk with Different Forage-to-Concentrate Diets

**DOI:** 10.3390/ani16071002

**Published:** 2026-03-24

**Authors:** Haiyun Yang, Sanshan Sun, Yusong Shen, Zhiwei Li, Changjiang Zang, Yong Chen, Fengming Li

**Affiliations:** 1 College of Animal Science, Xinjiang Agricultural University, Urumqi 830052, China; 2 Xinjiang Herbivore Nutrition Laboratory for Meat & Milk, Urumqi 830052, China

**Keywords:** diet, concentrate, roughage, camel milk, lipids

## Abstract

This study was conducted to investigate the effect of different concentrate-to-roughage ratios on the lipid composition of camel milk. Thirty-six Qiangar Bactrian camels were randomly allocated to three groups: the control group (C, grazing plus roughage only), the low-concentrate group (L, grazing plus roughage with 2 kg/d concentrate supplementation), and the high-concentrate group (H, grazing plus roughage with 4 kg/d concentrate supplementation). The trial comprised an 18-day adaptation period followed by a 42-day feeding period (60 days total). A systematic lipidomics approach revealed significant differences in milk lipid profiles among the three dietary treatments. High-concentrate diets markedly remodeled the milk lipidome (especially in positive-ion mode mass spectrometry), with the most pronounced differences detected between the C and H groups. Multivariate analyses (PCA and PLS-DA) confirmed that varying concentrate levels significantly affected milk lipid composition, and the separation between H and C was greater than that between L and C. KEGG annotation and enrichment analysis indicated that lipid-related pathways, notably glycerophospholipid metabolism, were significantly affected by dietary concentrate levels. These findings provide theoretical support for optimizing camel feeding strategies to improve milk nutritional quality and offer scientific guidance for camel husbandry and dairy product development.

## Simple Summary

This study investigated how different concentrate-to-roughage ratios affect the lipid composition of camel milk. Thirty-six Qiangar Bactrian camels were randomly allocated to three groups: the control group (C, grazing plus roughage only), the low-concentrate group (L, grazing plus roughage with 2 kg/d concentrate supplementation), and the high-concentrate group (H, grazing plus roughage with 4 kg/d concentrate supplementation). After 60 days, lipidomics analysis revealed significant differences in milk lipid profiles, particularly between the control and high-concentrate groups. Key lipid metabolic pathways, especially glycerophospholipid metabolism, were influenced by concentrate levels. These findings can help optimize camel feeding strategies to improve milk nutritional quality.

## 1. Introduction

As living standards continue to rise, consumer demand for dairy products has shifted from quantity to quality, with increasing attention paid to nutritional value and health benefits. Within this context, camel milk has garnered growing interest due to its unique composition, which includes a variety of bioactive substances and nutrients that distinguish it from conventional bovine milk [1]. Among its components, lipids play a particularly important role. The types and amounts of lipid components present in camel milk significantly influence its overall nutritional quality and functional properties [2]. Lipids are not only a major constituent of camel milk, contributing to its energy density and sensory characteristics, but they are also involved in numerous physiological functions, such as cell membrane formation, energy storage, and signal transduction [3]. A comprehensive understanding of the lipid composition of camel milk, as well as the factors influencing its variation, is therefore essential for optimizing production processes and enhancing the quality and commercial value of camel milk-derived products [4].

Diet is one of the most critical external factors affecting the composition of animal-derived foods. It is well established that dietary composition can markedly influence nutrient metabolism [5], alter rumen fermentation patterns [6,7,8], and subsequently affect the lipid profile and secretion of milk [9,10,11]. However, despite growing interest in camel milk, research specifically addressing the effects of different dietary regimes on its lipid composition remains limited. In particular, systematic lipidomic analyses investigating how varying dietary structures impact the camel milk lipidome are scarce. To address this knowledge gap, the present study employed a comprehensive lipidomic approach to explore how diets with different roughage-to-concentrate ratios alter the lipid profile of camel milk. By characterizing diet-induced changes in the milk lipidome, this research aims to provide theoretical insights that can support improved feeding management strategies and contribute to enhancing both the quality and yield of camel milk.

## 2. Materials and Methods

### 2.1. Experimental Design

Thirty-six Dzungar Bactrian camels with similar parity, body weight, and lactation days were selected from Urumqi, Xinjiang, and randomly divided into three groups (12 camels per group). All camels were raised by pasture-based feeding and supplementation. Camels in the control group (group C) were supplemented with roughage, and two treatment groups with roughage and 2 kg/d concentrate (group L) or 4 kg/d concentrate (group H), respectively. The experiment lasted for 60 days, consisting of an 18-day adaptation period and a 42-day formal feeding period, with milk samples collected on the last day of the formal period. The roughage mixture for group C was thoroughly mixed and ad libitum fed to the camels. For groups L and H, the roughage and concentrate were evenly mixed prior to feeding. The roughage mixture consisted of 80% wheat straw and 20% pasture forage, shredded to 3–4 cm. The concentrate composition is shown in Table 1.

On the last day of the trial, eight camels from each group were randomly selected for sampling; milk was collected using a dedicated milking device equipped with an automatic sampling tool. The udder was disinfected before each milking. Samples were aliquoted into sterile 5 mL cryotubes and stored in liquid nitrogen until analysis.

### 2.2. Measurement Indicators and Methods

#### 2.2.1. Main Instruments and Reagents

The main instruments and chromatographic column used in this study were as follows: Q-Exactive™ HF/HFX mass spectrometer, Vanquish™ UHPLC system (Thermo Fisher Scientific, Waltham, MA, USA), and Accucore C30 column (150 × 2.1 mm, 2.6 µm; Thermo Fisher Scientific, USA). Other auxiliary equipment include the Thermo ST16R high-speed centrifuge and a nitrogen blow-down concentrator (Reacti-Therm), among others. All instruments were sourced from Thermo in the USA.

This study employed various main reagents, including methanol (CH_3_OH), water (H_2_O), acetonitrile (CH_3_CN), formic acid (HCOOH), isopropanol (CH_3_CHOHCH_3_), ammonium acetate (CH_3_COONH_4_), and methyl tert-butyl ether (CH_3_OC(CH_3_)_3_), among others. All chemical reagents were purchased from Thermo Fisher Scientific (USA). The SPLASH™ lipid standard (Avanti Polar Lipids, Alabaster, AL, USA) was used as the internal standard for lipidomic analysis.

#### 2.2.2. Sample Extraction

100 μL of liquid milk sample and 0.75 mL of pre-chilled methanol (CH3OH) were added into a centrifuge tube with a PTFE-lined cap and vortexed. Then, 2.5 mL of pre-chilled methyl tert-butyl ether (C_5_H_12_O) was added and vortexed. Next, 10 μL of SPLASH™ internal standard was added and incubated in a shaker at room temperature for 1 h. 0.625 mL high-purity water was added, mixed, incubated at room temperature for 10 min, and centrifuge at 1000× *g* for 10 min. The upper organic phase (C_5_H_12_O) was collected. 1 mL of mixed solvent (C_5_H_12_O/CH_3_OH/H_2_O, 10:3:2.5, *v*/*v*/*v*) was added to the lower aqueous phase for a second extraction, then the organic phase was obtained. The organic phase was reconstituted with 100 μL of C_3_H_8_O, followed by LC-MS/MS detection and dissection. An equal volume (10 μL) of the supernatant from each processed sample was pooled to prepare quality control (QC) samples, which were analyzed while interspersed with experimental samples to evaluate analytical stability.

#### 2.2.3. Chromatographic Conditions

The column was the Thermo Accucore C30. The condition parameters were set as follows: temperature, 40 °C; flow rate, 0.35 mL/min; injection volume, 5 μL. Mobile phase A was acetonitrile:water (60:40, *v*/*v*) containing 0.1% formic acid and 10 mM ammonium acetate; mobile phase B was isopropanol:acetonitrile (90:10, *v*/*v*) containing 0.1% formic acid and 10 mM ammonium acetate.

f(CH_3_CN:H_2_O = 60:40) + 0.1% HCOOH + 10mMCH_3_COONH_4_; mobile phase B, (C_3_H_8_O:CH_3_CN = 90:10) + 0.1% HCOOH + 10mM CH_3_COONH_4_. The gradient elution procedure is shown in Table 2.

### 2.3. Data Analysis

First, all collected production index data were preprocessed (including data sorting and outlier removal) to ensure completeness and consistency. Subsequently, descriptive statistical methods were used to summarize and analyze each production index, calculating basic statistical measures such as the mean and standard deviation for each treatment group. To further evaluate the significance of differences between different treatment groups, one-way analysis of variance (one-way ANOVA) was employed for intergroup comparisons. If the ANOVA results indicated significant differences, Tukey’s HSD test was used for post hoc multiple comparisons to clarify the specific differences between groups. All statistical analyses were performed using SPSS 22.0 software, with the significance level set at *p* < 0.05.

Metabolomics data were processed with metaX to obtain VIP values for metabolites. 

Volcano plots were generated using the ggplot2 package in R software (v4.3.1). Differential lipids were identified using the following thresholds: variable importance in projection (VIP) > 1, |log2(fold change, FC)| > 0.263, and −log10(*p*-value) < 0.05. Clustering heat maps were generated using Origin, with z-score normalization applied to lipid data. Pearson correlation analysis among differential lipids was performed in R, and statistical significance was evaluated using cor.mtest, with *p* < 0.05 considered significant. Correlation plots were drawn using the R package corrplot.

Lipid identification and annotation used the Kyoto Encyclopedia of Genes and Genomes (KEGG) database (https://www.genome.jp/kegg/pathway.html, accessed on 14 March 2026), Human Metabolome Database (HMDB) (https://hmdb.ca/metabolites, accessed on 14 March 2026), and LIPID Maps, (http://www.lipidmaps.org/, accessed on 14 March 2026).

## 3. Results

### 3.1. Sample Quality Control

**Figure 1:**
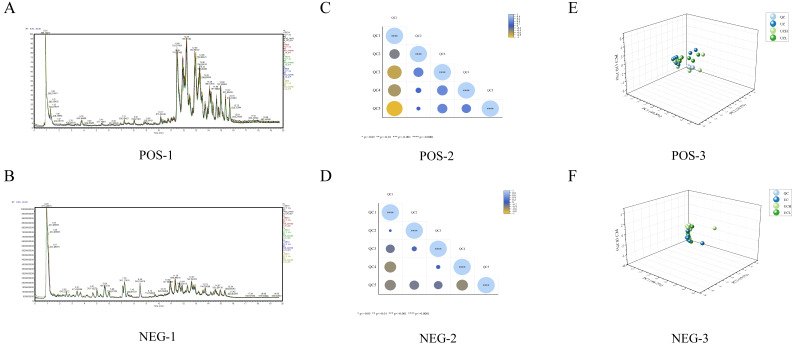
Quality control assessment of lipidomics analysis for camel milk samples. (**A**,**B**) Total ion chromatograms (TICs) in positive (**A**) and negative (**B**) ion modes; (**C**,**D**) Pearson correlation heatmaps of quality control (QC) samples; (**E**,**F**) 3D principal component analysis (PCA) score plots of all samples and QC samples.

Analytical stability was evaluated using quality control (QC) samples to verify the reliability of lipidomic analysis. Total ion chromatography from QC were overlaid for inspection (Figure 1A,B). As shown in Figure 1C,D, Pearson correlation coefficients among QCs were calculated from quantitative lipid values; high correlations indicate stable and reliable data acquisition. PCA of all samples and QCs showed tight clustering of QC injections, indicating method robustness and good data quality (Figure 1E,F). Thus, instrument performance, repeatability, and mass spectrometry data met the requirements for downstream analysis.

**Figure 2:**
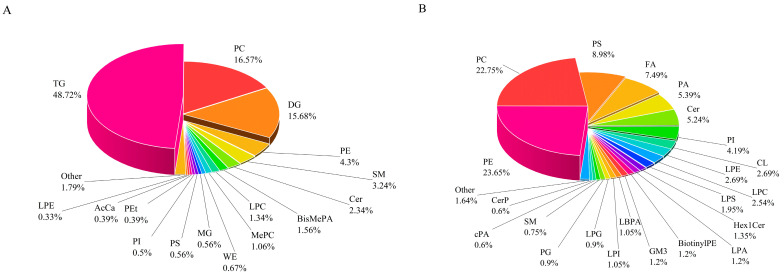
Classification of identified lipid species in positive (**A**) and negative (**B**) ion modes.

A total of 2460 lipid compounds were identified by combining positive and negative ion modes, with 1792 detected in positive ion mode and 668 in negative ion mode, respectively. In positive ion mode, the predominant lipid classes were triacylglycerols (TG, 48.72%), phosphatidylcholines (PC, 16.6%), diacylglycerols (DG, 15.7%) and phosphatidylethanolamines (PE, 4.3%). In negative ion mode, the dominant classes were PE (23.7%), PC (22.8%), phosphatidylserines (PS, 9%), and fatty acids (FA, 7.5%) (Figure 2).

**Figure 3:**
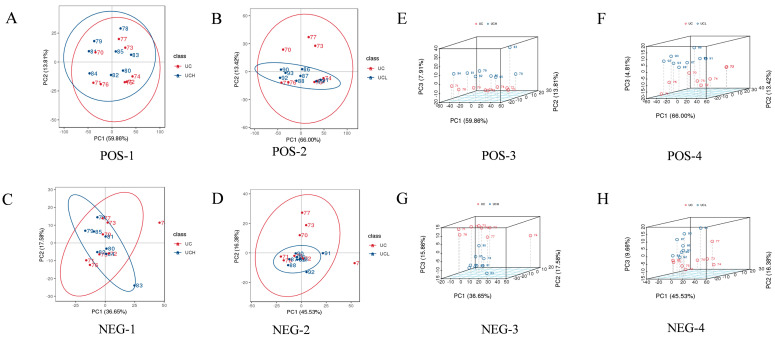
PCA and 3D PCA score plots of lipid compounds in camel milk under different concentrate-to-roughage ratios. (**A**–**D**) 2D PCA score plots in positive (**A**,**B**) and negative (**C**,**D**) ion modes; (**E**–**H**) 3D PCA score plots corresponding to 2D PCA.

For the comparison of C vs. H in positive ion mode (Figure 3A), PC1 explained 59.86% of the total variance and PC2 accounted for 13.81%, indicating that the intergroup differences were mainly driven by PC1. In negative ion mode for C vs. H, PC1 explained 36.65% of the variance and PC2 17.58%, respectively. For C vs. L in positive ion mode (Figure 3B), PC1 and PC2 explained 66.00% and 13.42% of the variance, respectively, also demonstrating that intergroup differences were primarily driven by PC1. In negative ion mode for C vs. L (Figure 3C), PC1 explained 45.53% of the variance and PC2 16.38%, again showing that PC1 was the main contributor to the intergroup differences.

The intergroup distribution was more intuitively observed via three-dimensional principal component analysis (3D PCA) plots, as shown in Figure 3. For C vs. H in positive ion mode (Figure 3E), PC1 explained 59.86% of the variance, PC2 13.81%, and PC3 7.91%. In negative ion mode for C vs. H (Figure 3G), PC1 explained 36.65% of the variance, PC2 17.58%, and PC3 15.86%. The 3D PCA plot revealed that the sample points of the H group were significantly separated from those of the C group along the PC1 and PC2 axes, indicating that the variation in concentrate-to-roughage ratio significantly affected milk lipid composition. For C vs. L in positive ion mode (Figure 3F), PC1 explained 66.00% of the variance, PC2 13.42%, and PC3 4.81%. In negative ion mode for C vs. L (Figure 3H), PC1 explained 45.53% of the variance, PC2 16.38%, and PC3 9.66%. The 3D PCA plot showed that the sample points of the L group were significantly separated from those of the C group along the PC1 and PC2 axes, which further confirmed the significant effect of concentrate-to-roughage ratio on milk lipid composition.

Through PCA and 3D PCA analyses, the following conclusions were drawn: significant differences in lipid composition were observed between the H group, L group, and C group, particularly along the PC1 axis. The degree of separation between the C group and H group was greater than that between the C group and L group, indicating that the addition of high levels of concentrate had a more significant effect on lipid composition. In positive ion mode, the separation between the C group and H group was mainly driven by PC1, while in negative ion mode, the separation between the C group and L group was more pronounced, suggesting that the expression patterns of lipid compounds differed across ion modes.

### 3.2. Orthogonal Partial Least Squares Discriminant Analysis (PLS-DA)

**Figure 4:**
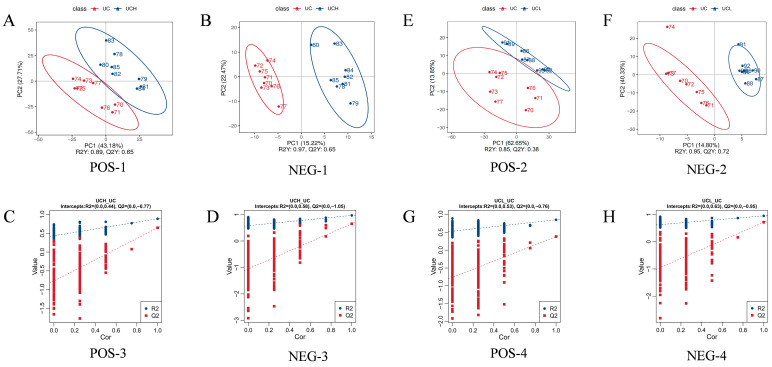
PLS-DA score plots and permutation test results of camel milk lipidomics data. (**A**,**B**,**E**,**F**) PLS-DA score plots for C vs. H (**A**,**B**) and C vs. L (**E**,**F**) in positive and negative ion modes; (**C**,**D**,**G**,**H**) permutation tests (*n* = 200) for validating model robustness (R^2^Y: explanatory ability; Q^2^Y: predictive ability).

Partial least squares discriminant analysis (PLS-DA), a supervised multivariate statistical method, was applied to enhance the discrimination among the three groups. Model performance is summarized in Figure 4. For C vs. H, the positive ion mode model (Figure 4A) showed R^2^Y = 0.89 and Q^2^ = 0.65; the negative ion mode model (Figure 4B) showed R^2^Y = 0.97 and Q^2^ = 0.65. For C vs. L, the positive ion mode model (Figure 4E) showed R^2^Y = 0.85 and Q^2^ = 0.38; the negative ion mode model (Figure 4F) showed R^2^Y = 0.95 and Q^2^ = 0.72.

The PLS-DA models for the C vs. H comparison (Figure 4C,D) exhibited excellent explanatory power (R^2^Y) and predictive ability (Q^2^) in both positive and negative ion modes. The L vs. C model had lower predictability in the positive ion mode (Figure 4G) but performed well in the negative ion mode (Figure 4H). Permutation testing indicated no overfitting (the intercepts of R^2^Y and Q^2^ with the abscissa were less than 1).

**Figure 5:**
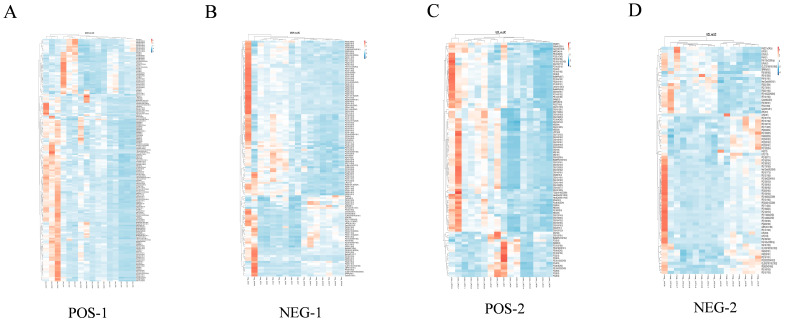
Hierarchical clustering heatmaps of differentially expressed lipid metabolites. (**A**) H vs. C (positive ion mode); (**B**) H vs. C (negative ion mode); (**C**) L vs. C (positive ion mode); (**D**) L vs. C (negative ion mode). The color scale represents standardized expression levels (z-score).

Differential lipids were screened using the criteria of VIP > 1 and *p* < 0.05 (one-way ANOVA), followed by compound identification and Benjamini–Hochberg false discovery rate (FDR) correction (*p* < 0.05). This process identified 1683 and 654 differential lipids in the positive and negative ion modes, respectively, and their accumulation patterns were visualized by hierarchical clustering heatmaps (Figure 5). Differential accumulation patterns were visualized using clustered heat maps (Figure 5).

### 3.3. Differential Lipids

Based on the predefined screening criteria (VIP > 1.0, |log_2_FC| > 0.263, *p* < 0.05), differential lipid compounds were further identified among the groups.

**Figure 6:**
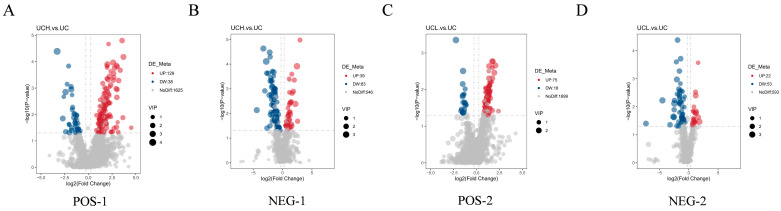
Volcano plots of differential lipid metabolites between treatment and control groups. (**A**) H vs. C (positive mode); (**B**) H vs. C (negative mode); (**C**) L vs. C (positive mode); (**D**) L vs. C (negative mode). Red: upregulated (VIP > 1.0, *p* < 0.05, log_2_FC > 1); blue: downregulated (VIP > 1.0, *p* < 0.05, log_2_FC < −1); gray: non-significant.

A total of 289 differential lipids were identified in the C vs. H comparison, including 167 in positive ion mode (129 upregulated, 38 downregulated) and 122 in negative ion mode (39 upregulated, 83 downregulated) (Table 3, Figure 6). For C vs. L, 168 differential lipids were identified (93 in positive ion mode: 75 upregulated, 18 downregulated; 75 in negative ion mode: 22 upregulated, 53 downregulated) (Table 3).

**Figure 7:**
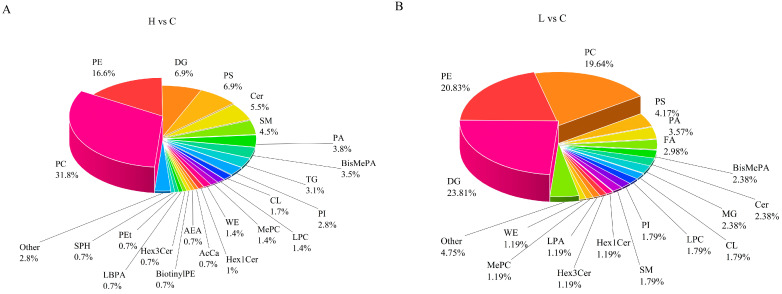
Distribution of lipid classes for differentially expressed lipids. (**A**) H vs. C; (**B**) L vs. C. Percentages indicate the proportion of each lipid class in the total differential lipids.

In the H vs. C comparison (combined ion modes), 30 lipid classes were significantly altered, with phosphatidylcholine (PC), phosphatidylethanolamine (PE), and phosphatidylserine (PS) being the top three affected classes (Figure 7A). Among the 92 differentially regulated PC lipids, 29 (31.52%) were significantly upregulated (e.g., PC 14:0/15:2, PC 15:0/19:4), and 63 (68.48%) were significantly downregulated (e.g., PC 12:1/18:3, PC 14:0/15:0). 48 PEs were differentially regulated, with 24 upregulated ones (e.g., PE 14:0/16:1, PE 14:0/18:2), and 24 showing other regulation patterns.

For L vs. C (combined modes), 26 lipid categories were affected, including DG, PE, PC, PS, PA, FA, BisMePA, Cer, MG, CL, and LPC (Figure 7B). Forty DG lipids were upregulated (e.g., DG 10:0/12:0, DG 10:0/16:1), while two DG lipids (DG 13:0/18:1 and DG 19:2) were downregulated. Among 35 PE lipids, nine were upregulated (e.g., PE 14:0/18:2, PE 16:0/18:2) and 26 were downregulated (e.g., PE 12:0/17:0, PE 14:0/15:0). Detailed compound lists are provided in Supplementary Table S1.

### 3.4. KEGG Pathway Annotation and Enrichment Analysis of Differential Lipid Compounds in Each Comparison Group

**Figure 8:**
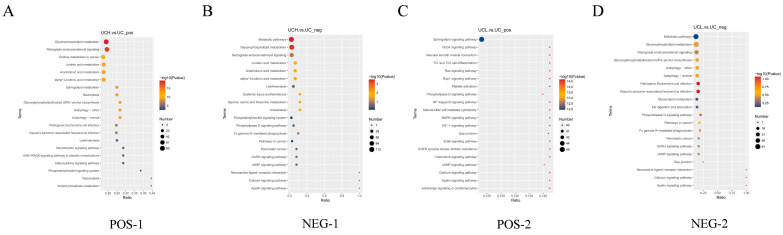
KEGG pathway enrichment analysis of differential lipid compounds in positive and negative ion modes. (**A**) H vs. C (positive); (**B**) H vs. C (negative); (**C**) L vs. C (positive); (**D**) L vs. C (negative). The bubble size represents the number of differential lipids, and the color represents the *p*-value of hypergeometric test.

Kyoto Encyclopedia of Genes and Genomes (KEGG) pathway annotation and enrichment analysis was performed to explore the metabolic pathways associated with differential lipid compounds. The enrichment degree was calculated as the ratio of the number of differential lipids to the total identified lipids in each pathway, and the hypergeometric test was used to determine the statistical significance (*p*-value). The top 20 enriched pathways were visualized as bubble plots (Figure 8), where the *x*-axis indicated enrichment ratio, bubble color represented *p*-value (darker color for lower *p*-value), and bubble size denoted the number of differential lipids in each pathway. Notably, glycerophospholipid metabolism was the most significantly enriched pathway in negative ion mode for the H vs. C comparison, which was the core lipid metabolic pathway affected by the high-concentrate diet. In the H vs. C comparison, differential lipids were significantly enriched in glycerolipid metabolism and retrograde endocannabinoid signaling pathways in positive ion mode, whereas metabolic pathways, glycerophospholipid metabolism, and glycine-serine-threonine metabolism were significantly enriched in negative ion mode (Figure 8A,B). For L vs. C, EGFR tyrosine kinase inhibitor resistance was the main enriched pathway in positive ion mode, and pathogenic Escherichia coli infection and Kaposi’s sarcoma-associated herpesvirus infection were enriched in negative ion mode (Figure 8C,D).

### 3.5. Screening of Characteristic Lipid Compounds in Camel Milk

**Figure 9:**
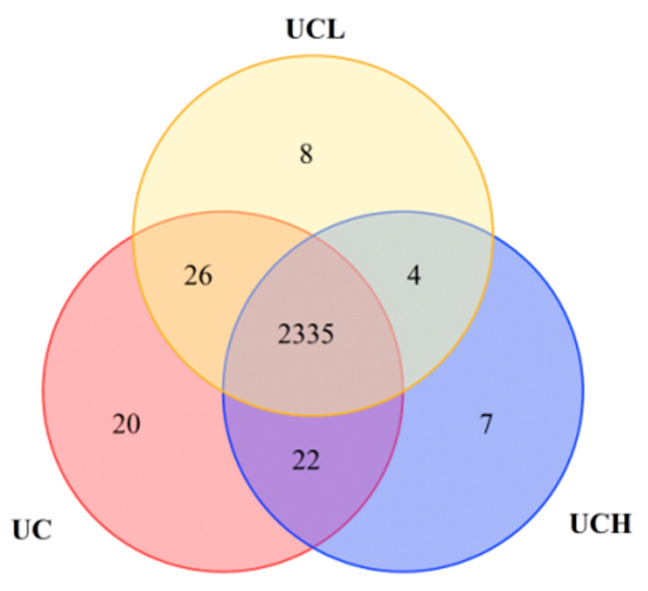
Venn diagram showing the overlap and uniqueness of lipid compounds across C, L, and H groups. Numbers indicate the count of unique or shared lipid compounds among groups.

To identify unique lipid compounds and screen for specific lipid markers in camel milk under different concentrate-to-roughage ratios, lipid compounds detected by qualitative and quantitative analysis were retained using a missing value threshold of 50% (i.e., a compound was considered present if detected in at least 50% of the samples in each group). A Venn diagram was used to show the overlap and uniqueness of lipid compounds among the C, L, and H groups (Figure 9), and a greater number of unique lipid compounds were identified in the positive ion mode compared with the negative ion mode.

Table 4 presents the characteristics of seven lipid compounds that were uniquely identified in the H group. The lipids are categorized into three classes: Ceramides (Cer), Lysobisphosphatidic acid (LBPA), and Triacylglycerols (TG). For each compound, the mode of ionization (positive or negative) and the chemical formula are provided. Among these, three ceramides—Cer (d12:0/23:0), Cer (d16:0/18:0), and Cer (m18:1/16:0)—were detected in positive ion mode. One LBPA species, LBPA (14:0/18:2), was detected in negative ion mode. Additionally, three triacylglycerols—TG (10:0CHO/12:0/12:0), TG (18:2CHO/8:0/10:0), and TG (4:0CHO/14:0/16:0)—were detected in positive ion mode. These lipid species were found exclusively in the H group, suggesting their potential role as biomarkers or indicators of normal physiological conditions.

Table 5 summarizes the characteristics of eight lipid compounds uniquely identified in the low-concentrate group (L). The lipids are organized into two main classes: Lysophosphatidylcholine (LPC) and Triacylglycerols (TG). For each compound, the ionization mode and chemical formula are specified. One LPC species, LPC (18:0), was detected in negative ion mode. The remaining seven lipids are triacylglycerols, all detected in positive ion mode, including TG (18:0COOH/15:0/16:0), TG (19:2/4:0/15:1CHO), TG (19:4/4:0/6:0), TG (4:0/5:0/18:1), TG (4:1CHO/12:0/16:1), and TG (6:0/9:0/12:0). These lipid species were found exclusively in the low-concentrate group, indicating their potential relevance as metabolic markers associated with this dietary condition.

## 4. Discussion

### 4.1. Effect of Dietary Concentrate-to-Roughage Ratios on Milk Composition

Changes in the concentrate-to-roughage ratios (groups C, L, H) altered the camel milk metabolome. PCA (Figure 2 and Figure 3) showed clear separation between group C and group H in both ion modes; PC1 accounted for the majority of variance (positive mode: 59.86% for C vs. H; negative mode: 66.00%). The separation between C and H was further than between C and L, indicating a stronger metabolic effect with a higher concentrate inclusion.

Different concentration-to-roughage ratios affect rumen microbial composition and metabolic activity [12]. High-concentrate diets increase the content of starch and carbohydrates in the rumen [13,14], favoring amylolytic bacteria [15] and increasing the production of volatile fatty acids (VFAs) [16]. Low-concentrate diets favor fibrolytic bacteria [17], producing more acetic and butyric acids [18], which can support higher milk fat content [19].

Dietary shifts also change milk metabolite profiles [10] and the contents of milk [20]. High-concentrate diets have been associated with increased ethanol [21] and lactic acid [22], whereas low-concentrate diets may increase certain amino acids [23] and VFAs [24,25].

Adjusting concentrate-to-roughage ratios produced significant changes in milk lipid type and abundance [26]. High-concentrate diets may increase dietary unsaturated fatty acids, potentially raising milk polyunsaturated fatty acid proportions [27]. Studies reported increased milk oleic acid [28] and linoleic acids [29] with higher concentrate feeding. Triglyceride and phospholipid contents may also change with increasing concentrate-to-roughage ratios, reflecting alterations in fat synthesis and secretion [30,31]. Dietary concentrate-to-roughage ratios alter the composition of milk fat by affecting lipid metabolism pathways in ruminants [32]. High-concentrate feeding can modify rumen fermentation, decreasing acetic acid production [33] and thus affecting the short-chain fatty acid synthesis in milk fat [34]. Rumen biohydrogenation by microorganisms may also alter the unsaturated fatty acid profils in milk fat [35].

### 4.2. Relationships of Lipid Differential Compounds

Diets with different concentrate-to-roughage ratios lead to different milk lipid compositions [11,36]. In C vs. H, differential lipids spanned 30 catagories (PC, PE, PS, DG, Cer, SM, PA, BisMePA, TG, PI, CL, etc.). Among them, PC (phosphatidylcholine) and PE (phosphatidylethanolamine) are major components of cell membranes [37], which are involved in physiological processes, such as the maintenance of cell structure [38,39,40] and material transport [41], and play a key role in milk secretion [42,43,44,45]. DG (diacylglycerol) is a precursor for triacylglycerol [46,47] and phospholipid [48] synthesis, and is involved in energy storage [49] and cell signaling [50]. Cer (ceramides) participate in apoptosis, differentiation, proliferation [51,52,53,54], barrier function [55,56], and mucous membranes [57]. SM (sphingomyelin) plays a PC-like role in the structure and function of cell membranes and is also involved in processes such as cell signaling and nerve conduction [58].

High-concentrate diets were associated with decreased milk fat percentage in some contexts [59,60] due to altered rumen fermentation patterns [12,61,62] and lower acetate production, a key precursor to milk fat synthesis [9,63,64,65]. The fiber in roughage can promote rumen fermentation [66], increase acetic acid production, and maintain a stable milk fat rate [64].

The concentrate-to-roughage ratio also has an effect on fatty acid composition [11,67]. The high concentrate diet led to a decrease in saturated fatty acid content [68], which may be due to higher ruminal hydrogenation of unsaturated fatty acids to saturated fatty acids [69]. Oleic acid content, which is the main component of monounsaturated fatty acids [28,70], may change depending on ruminal biohydrogenation rate rates [71,72].

### 4.3. Interpretation of Pathway Results

This study demonstrates that diets with different concentrate-to-roughage ratios significantly influence milk lipid composition [11,73], especially when concentrate inclusion is high. Multivariate statistical analyses (PCA and PLS-DA) showed greater separation between the high-concentrate group and control group than between the medium-concentrate group and control [74]. KEGG enrichment implicated pathways such as glycerophospholipid metabolism, suggesting that dietary ratios may alter lipid synthesis, degradation, and transformation pathways [75] that determine milk lipid composition and quality.

## 5. Conclusions

This study applied lipidomics to examine the effects of different concentrate-to-roughage ratios on camel milk lipid composition. High concentrate addition significantly altered the milk lipidome, with the greatest differences observed between the control and high-concentrate groups. Multivariate analyses confirmed that the high-concentrate diet produced more pronounced changes than the medium-concentrate diet. KEGG enrichment highlighted significant alterations in pathways such as glycerophospholipid metabolism. These findings provide theoretical support for optimizing camel feeding strategies to improve milk lipid quality and offer a basis for future studies on camel feeding management and dairy development.

## Figures and Tables

**Table 1:** Composition and nutritional levels of concentrated feed (on a dry matter basis).

Ingredients	Contents (%)	Nutritional Composition ^2^	Contents (%)
Corn	58.50	Dry matter (DM)	94.56
Wheat bran	9.00	Crude protein (CP)	15.62
Soybean meal	18.00	Crude fiber (CF)	3.55
Cottonseed meal	5.75	Calcium (Ca)	0.97
Fat powder	3.50	Phosphorus (P)	0.53
NaCl	2.00	Gross energy GE, MJ/kg	17.46
Premix ^1^	1.00		
Limestone	1.50		
CaHPO_4_	0.75		
Total	100.00		

^1^ The premix provides the following per kilogram of concentrate: FeSO_4_: 135.00 mg, KI: 1.50 mg, MnSO_4_: 60.00 mg, ZnSO_4_: 60.00 mg, CuSO_4_: 15.00 mg, Vitamin A: 3600 IU, Vitamin D: 396 IU, Vitamin E: 1200 IU, Vitamin B_1_: 4.50 mg, Vitamin B_2_: 3.00 mg. ^2^ The nutritional composition are measured values.

**Table 2:** Chromatographic gradient elution procedure for lipidomics analysis.

Time (min)	A %	B %
initial	70	30
2	70	30
5	57	43
5.1	45	55
11	30	70
16	1	99
18	1	99
18.1	70	30

**Table 3:** Statistics of differential lipid compounds under different concentrate-to-roughage ratios.

Grouping	Total Lipid Compounds			Significant Upward Revision			Significant Downward Revision		
	Total	Pos	Neg	Total	Pos	Neg	Total	Pos	Neg
C vs. H	289	167	122	168	129	39	121	38	83
C vs. L	168	93	75	97	75	22	71	18	53

**Table 4:** Characteristics of seven unique lipid compounds in the control group (H).

Lipid Compounds	Mode	Chemical Formula
Ceramides (3)		
Cer (d12:0/23:0)	pos	C_35_H_70_NO_2_
Cer (d16:0/18:0)	pos	C_34_H_68_NO_2_
Cer (m18:1/16:0)	pos	C_34_H_67_NO_2_
Lysophosphatidic acid (1)		
LBPA (14:0/18:2)	neg	C_32_H_64_NO_6_P_2_
Triacylglycerol (19)		
TG (10:0CHO/12:0/12:0)	pos	C_37_H_70_O_4_
TG (18:2CHO/8:0/10:0)	pos	C_39_H_75_O_4_
TG (4:0CHO/14:0/16:0)	pos	C_37_H_74_O_4_

**Table 5:** Characteristics of eight unique lipid compounds in the low-concentrate group (L).

Lipid Compounds	Mode	Chemical Formula
Lysophosphatidylcholine (1)		
LPC (18:0)	neg	C_26_H_56_NO_8_P
Triacylglycerol (7)		
TG (18:0COOH/15:0/16:0)	pos	C_49_H_90_O_7_
TG (19:2/4:0/15:1CHO)	pos	C_48_H_82_O_5_
TG (19:4/4:0/6:0)	pos	C_39_H_64_O_6_
TG (4:0/5:0/18:1)	pos	C_27_H_52_O_6_
TG (4:0/6:0/17:1)	pos	C_27_H_52_O_6_
TG (4:1CHO/12:0/16:1)	pos	C_32_H_58_O_5_
TG (6:0/9:0/12:1)	pos	C_27_H_52_O_6_

## Data Availability

Data will be made available on request.

## Supplementary Materials

The following supporting information can be downloaded at: https://www.mdpi.com/article/10.3390/ani16071002/s1. Detailed compound lists are provided in Supplementary Table S1.

## Abbreviations

The following abbreviations are used in this manuscript:

C	Control group
L	Low-concentrate group
H	High-concentrate group
PCA	Principal Component Analysis
3D PCA	Three-Dimensional Principal Component Analysis
PLS-DA	Partial Least Squares Discriminant Analysis
VIP	Variable Importance in Projection
FC	Fold change
QC	Quality control
TIC	Total Ion Chromatogram
KEGG	Kyoto Encyclopedia of Genes and Genomes
HMDB	Human Metabolome Database
LC-MS/MS	Liquid Chromatography-Tandem Mass Spectrometry
UHPLC	Ultra-High Performance Liquid Chromatography
TG	Triacylglycerol
DG	Diacylglycerol
PC	Phosphatidylcholine
PE	Phosphatidylethanolamine
PS	Phosphatidylserine
PI	Phosphatidylinositol
PA	Phosphatidic acid
CL	Cardiolipin
Cer	Ceramide
SM	Sphingomyelin
LPC	Lysophosphatidylcholine
FA	Fatty acid
BisMePA	Bis(methyl)phosphatidic acid
VFA	Volatile fatty acid
FDR	False discovery rate
ANOVA	Analysis of variance
HSD	Honestly significant difference
PTFE	Polytetrafluoroethylene
EGFR	Epidermal Growth Factor Receptor
