# Supplementary material for: Study on Differences in Lipid Composition of Camel Milk with Different Forage-to-Concentrate Diets
Source: Animals (Basel). 2026 Mar 24;16(7):1002. doi: 10.3390/ani16071002 (PMC13072413; doi:10.3390/ani16071002)
Supplement: Supplementary file 1 [file animals-16-01002-s001.zip › animals-4132820-supplementary.pdf]

Table S1. Detailed compound lists are provided

Name	Pattern	Class	FC	Pvalue	VIP	Up.Down
Acylcarnitine（2）						
AcCa(4:0)	pos	AcCa	2.962465756	0.012986475	1.665564387	up
AcCa(5:0)	pos	AcCa	2.165067771	0.03778202	1.183005361	up
N-Neurocerebroside（2）						
AEA(13:0)	pos	AEA	1.967444501	0.045759614	1.009934168	up
AEA(16:0)	pos	AEA	2.021085818	0.021908506	1.357393374	up
Biotinylated phosphatidylethanolamine（2）						
BiotinylPE (29:6)	neg	BiotinylPE	2.093762068	0.01954635	2.228902039	up
BiotinylPE (31:7)	neg	BiotinylPE	2.153262033	0.038153356	2.206834682	up
Dimethylphosphatidic acid（14）						
BisMePA(18:2/18:3)	pos	BisMePA	4.58249455	0.011810116	1.416778065	up
BisMePA(18:3/12:0)	pos	BisMePA	7.792648181	0.00013889	2.335732554	up
BisMePA(18:3/12:0)	pos	BisMePA	2.28621153	0.014295069	1.079267932	up
BisMePA(18:4/16:1)	pos	BisMePA	6.706283577	0.000175211	2.292041788	up
BisMePA(18:4/18:1)	pos	BisMePA	6.520776992	0.03314346	1.73542951	up
BisMePA(32:5)	pos	BisMePA	4.506516995	0.001671292	1.535457091	up
BisMePA(33:3)	pos	BisMePA	3.583616271	0.004747103	1.113750966	up
BisMePA(34:6)	pos	BisMePA	12.99711043	6.62892E-05	2.741937782	up
BisMePA(34:6)	pos	BisMePA	2.542127199	0.012293714	1.255293521	up
BisMePA(36:7)	pos	BisMePA	11.78573808	1.59096E-05	2.261698009	up
BisMePA(36:7)	pos	BisMePA	2.702825646	0.006282125	1.118679927	up
BisMePA(36:8)	pos	BisMePA	8.205354086	0.001143836	1.932803739	up
BisMePA(38:7)	pos	BisMePA	5.103107561	0.000763017	2.410239152	up
BisMePA(10:0/16:2)	pos	BisMePA	0.339428057	0.042596879	1.060351669	down
Ceramide（20）						

Cer(d12:0/18:0)	pos	Cer	2.678799908	0.013560813	1.542304608	up
Cer(d12:0/20:0)	pos	Cer	2.625931833	0.034657954	1.370736153	up
Cer(d12:0/22:0)	pos	Cer	3.404264623	0.001541026	1.465508487	up
Cer(d16:1/22:3)	pos	Cer	7.837667643	0.030028889	1.939776398	up
Cer(d16:1/22:3)	pos	Cer	3.447911503	0.001679456	1.906926718	up
Cer(d18:1/21:3)	pos	Cer	2.50047173	0.009162643	1.44213888	up
Cer(d18:2/23:0)	pos	Cer	2.323634753	0.009861955	1.039638216	up
Cer(d19:1/18:0)	pos	Cer	2.378752262	0.002782046	1.460215324	up
Cer(d19:1/23:3)	pos	Cer	3.217841159	0.011620893	1.538862404	up
Cer(d20:1/23:3)	pos	Cer	2.395576752	0.02520333	1.169476506	up
Cer(m18:0/16:0)	pos	Cer	1.796879453	0.021051005	1.141604485	up
Cer(m18:0/22:0)	pos	Cer	3.152987257	0.013191441	1.85259862	up
Cer(m34:2)	pos	Cer	2.304509705	0.029875181	1.558410519	up
Cer(t20:1/23:2)	pos	Cer	2.333107634	0.031707112	1.107958086	up
Cer(d18:0/16:0)	neg	Cer	1.645633094	0.028776226	1.64490527	up
Cer(d18:2/20:0)	neg	Cer	1.492007128	0.031520358	1.683554211	up
Cer(d18:2/22:0)	neg	Cer	1.522147961	0.038867273	1.346697463	up
Cer(d18:2/24:1)	neg	Cer	1.427799608	0.030688058	1.516745096	up
Cer(d19:1/24:1)	neg	Cer	1.984110783	0.010068718	1.016702606	up
Cer(d19:1/24:1)	neg	Cer	1.677219442	0.014893138	1.117275097	up
Phosphatidylcholine（1）						
CerP(d40:1)	neg	CerP	1.954265946	0.012507978	2.11270208	up
Cardiolipin（8）						
CL(22:1CHO/18:0/22:5/22:5)	neg	CL	2.531936871	0.035716333	1.616427248	up
CL(27:3/16:0/16:0/18:0)	neg	CL	5.487842301	0.000122379	3.1092887	up
CL(27:3/16:0/16:0/18:0)	neg	CL	2.279968493	0.003989029	2.58751175	up
CL(42:5CHO/16:0/18:1)	neg	CL	2.224117558	0.004330947	1.559558361	up
CL(18:2/18:2/18:2/18:2)	neg	CL	0.461231468	0.02661532	2.561385593	down
CL(24:2/18:1/18:2/18:2)	neg	CL	0.24761229	0.048511745	2.255764385	down

CL(45:9CHO/19:0/18:1)	neg	CL	0.283592078	0.000487762	2.244380031	down
CL(69:14CHO/17:0)	neg	CL	0.264197501	0.000488505	1.313098046	down
Cyclic phospholipid acid（2）						
cPA(16:1)	neg	cPA	2.121037436	0.01700648	1.4888677	up
cPA(18:0)	neg	cPA	1.644464471	0.030481587	2.067815566	up
Diacylglycerol（60）						
DG(10:0/12:0)	pos	DG	2.132541364	0.026473881	1.082745989	up
DG(10:0/16:1)	pos	DG	2.403765724	0.021536194	1.304159571	up
DG(10:0/27:7)	pos	DG	1.891609687	0.022570792	1.271510884	up
DG(12:0/14:0)	pos	DG	2.667202689	0.014946655	1.013664765	up
DG(12:0/18:1)	pos	DG	2.472981575	0.043916048	1.369959453	up
DG(12:0/18:2)	pos	DG	2.721089752	0.012199649	1.245684262	up
DG(12:0/20:5)	pos	DG	2.872243169	0.010738543	1.249278	up
DG(14:0/16:1)	pos	DG	2.604885115	0.014319444	1.488713566	up
DG(14:0/18:1)	pos	DG	2.576537476	0.011265273	1.533209109	up
DG(14:0/18:2)	pos	DG	3.684711805	0.022248091	1.555076228	up
DG(14:0/18:2)	pos	DG	3.866118264	0.003446625	2.171223816	up
DG(14:0/20:5)	pos	DG	2.59012525	0.011669777	1.3780319	up
DG(15:2/17:0)	pos	DG	2.485009079	0.022150664	1.051948283	up
DG(16:0)	pos	DG	2.834762395	0.006259847	1.679259078	up
DG(16:0/14:2)	pos	DG	2.290378351	0.018227213	1.379879422	up
DG(16:0/16:3)	pos	DG	1.928517346	0.031415365	1.023476207	up
DG(16:0/18:0)	pos	DG	1.977506644	0.00897536	1.102758864	up
DG(16:0/18:2)	pos	DG	2.166201604	0.015402884	1.194802339	up
DG(16:0/18:4)	pos	DG	2.071998178	0.018195423	1.290231768	up
DG(16:0/20:3)	pos	DG	1.997805425	0.009255746	1.091822618	up
DG(16:0/20:4)	pos	DG	2.203980172	0.01131294	1.36046058	up
DG(16:0/24:6)	pos	DG	2.792847915	0.032974809	1.181298618	up
DG(16:1/16:0)	pos	DG	3.647387991	0.004291248	2.44916502	up
DG(16:1/16:0)	pos	DG	2.143166636	0.02368553	1.649343459	up

DG(16:1/16:1)	pos	DG	2.674597709	0.011562009	1.38213522	up
DG(16:1/18:4)	pos	DG	3.060637343	0.048183307	1.260116952	up
DG(16:1/18:4)	pos	DG	3.335883952	0.004858412	1.895021595	up
DG(16:1/22:5)	pos	DG	2.670252462	0.010555662	1.376082411	up
DG(16:3/16:1)	pos	DG	8.035886123	0.002239133	2.277939685	up
DG(16:3/16:1)	pos	DG	2.319852045	0.011387643	1.330054561	up
DG(17:1/20:0)	pos	DG	2.460422726	0.019587851	1.458298714	up
DG(17:2/15:1)	pos	DG	1.81757411	0.010720421	1.388179345	up
DG(17:2/15:2)	pos	DG	2.519748296	0.023881446	1.655438031	up
DG(17:2/15:2)	pos	DG	2.072882468	0.043716251	1.305526263	up
DG(17:2/20:4)	pos	DG	2.019416383	0.011765446	1.047352443	up
DG(17:2/23:6)	pos	DG	2.101437702	0.037082836	1.835131154	up
DG(18:1/20:1)	pos	DG	1.8036264	0.028895546	1.127189138	up
DG(18:1/22:6)	pos	DG	2.245410948	0.034694295	1.211559792	up
DG(18:2/20:4)	pos	DG	3.3310092	0.002501819	1.267906687	up
DG(18:4/12:0)	pos	DG	2.137646124	0.028904702	1.186976506	up
DG(18:4/15:2)	pos	DG	2.179797865	0.030473477	1.174610757	up
DG(18:4/18:0)	pos	DG	2.217240229	0.015800447	1.760791829	up
DG(23:6/13:0)	pos	DG	2.91245194	0.004967559	1.900255213	up
DG(24:6/16:0)	pos	DG	2.843560126	0.032222629	1.188152529	up
DG(26:4)	pos	DG	2.962871251	0.002189954	1.939813447	up
DG(26:4)	pos	DG	2.070358606	0.026533814	1.265635054	up
DG(28:7)	pos	DG	1.946147906	0.045938589	1.080354744	up
DG(4:0/16:1)	pos	DG	2.36228156	0.019393149	1.141684008	up
DG(5:0/6:0)	pos	DG	3.665224054	0.011504462	1.733957998	up
DG(8:0/18:4)	pos	DG	2.490650393	0.019081345	1.389837514	up
DG(13:0/15:0)	pos	DG	0.295533957	0.032138619	2.021617347	down
DG(13:0/18:1)	pos	DG	0.363099503	0.022261242	1.633592595	down
DG(17:0/18:0)	pos	DG	0.43106993	0.011135005	1.527376727	down

DG(18:0/20:2)	pos	DG	0.287469679	0.040208365	1.945675953	down
DG(18:0/22:5)	pos	DG	0.48267265	0.047080726	1.307664097	down
DG(18:2/20:0)	pos	DG	0.498446422	0.035341353	1.131102767	down
DG(19:2)	pos	DG	0.411137023	0.022354315	1.970650737	down
DG(19:3/18:0)	pos	DG	0.429481641	0.010501172	1.534059518	down
DG(20:0/18:1)	pos	DG	0.245836376	0.024591094	1.517944241	down
DG(20:0/20:4)	pos	DG	0.242754935	0.023093928	1.371662684	down
Fatty acid（6）						
FA(16:1)	neg	FA	2.009898791	0.034986468	1.407770425	up
FA(17:0)+COOH:(s)	neg	FA	1.838903577	0.04540895	1.352546907	up
FA(22:1)+OX:(s)	neg	FA	3.030519385	0.023668804	2.473655077	up
FA(14:0)+COOH:(s)	neg	FA	0.15979913	0.039274414	2.02071707	down
FA(14:0)+COOH:(s)	neg	FA	0.110115338	0.043975992	2.390738372	down
FA(27:3)	neg	FA	0.429912253	0.022468056	2.253859181	down
Ganglioside GM3（1）						
GM3(d36:1)	neg	GM3	2.800701893	0.003295576	1.543642952	up
Monosaccharide ceramide（5）						
Hex1Cer(d18:1/24:1)	pos	Hex1Cer	3.320316523	0.000975019	1.724839832	up
Hex1Cer(d19:1/22:0)	pos	Hex1Cer	8.539822658	0.019748646	1.03102097	up
Hex1Cer(d19:0/18:1)	neg	Hex1Cer	1.93935237	0.033950904	1.56617259	up
Hex1Cer(t18:1/18:1)	neg	Hex1Cer	2.242756976	0.009025019	1.690246524	up
Hex1Cer(d18:2/24:0)	neg	Hex1Cer	0.183959332	0.005903206	2.012919906	down
Trihexosyl-ceramide（4）						
Hex3Cer(d18:1/16:0)	pos	Hex3Cer	4.062920634	0.011180927	1.444984461	up
Hex3Cer(d18:1/16:0)	pos	Hex3Cer	2.71020805	0.041929498	1.082765269	up
Hex3Cer(d18:1/22:0)	pos	Hex3Cer	4.861768749	0.00364675	1.722683135	up
Hex3Cer(d18:1/22:0)	pos	Hex3Cer	2.914864765	0.017339471	1.34393708	up
Hemolytic phosphatidic acid（3）						
LBPA(14:0/16:1)	neg	LBPA	5.924818473	0.020690124	1.744699937	up

LBPA(16:1/16:1)	neg	LBPA	1.66778087	0.021892269	1.445744949	up
LBPA(18:1/18:2)	neg	LBPA	0.163657179	0.022605703	3.319662391	down
Lysophosphatidic acid（2）						
LPA(16:1)	neg	LPA	1.599961296	0.042379448	1.44176906	up
LPA(18:3)	neg	LPA	0.391179675	0.038845715	1.399803573	down
Lyso-phosphatidylcholine（7）						
LPC(16:2)	pos	LPC	2.37317487	0.04647016	1.560776645	up
LPC(16:2)	pos	LPC	2.393115122	0.009591866	1.862881601	up
LPC(28:1)	pos	LPC	3.07518209	0.007799543	1.709314122	up
LPC(28:2)	pos	LPC	2.231404389	0.046791533	1.269191997	up
LPC(15:0)	neg	LPC	0.452380609	0.023810152	2.139857765	down
LPC(17:0)	neg	LPC	0.442884942	0.015347814	2.556521917	down
LPC(17:0)	neg	LPC	0.453571134	0.025292794	1.788378307	down
Lipopolysaccharide（1）						
LPG(18:1)	neg	LPG	0.006400797	0.039744503	2.553098335	down
Hemolytic phosphatidylinositol（1）						
LPI(16:1)	neg	LPI	2.288250696	0.035126202	1.361623139	up
Lipopolysaccharide（2）						
LPS(20:0)	pos	LPS	2.954181318	0.045479451	1.034562447	up
LPS(16:1)	neg	LPS	1.828475638	0.032891349	1.507667293	up
Methylphosphatidylcholine（6）						
MePC(32:4)	pos	MePC	3.64432363	0.019509508	1.562043382	up
MePC(39:10)	pos	MePC	6.10302001	0.000107934	2.888069721	up
MePC(39:10)	pos	MePC	3.301540675	0.001695691	2.257749144	up
MePC(41:11)	pos	MePC	4.364163416	2.15192E-05	1.267554742	up
MePC(46:14)	pos	MePC	3.417429144	0.003194343	2.198806996	up
MePC(46:14)	pos	MePC	1.863034026	0.037318989	1.634395316	up
Monogalactosyl diacylglycerol（5）						

MG(16:0)	pos	MG	2.759406122	0.007647835	1.625429371	up
MG(16:1)	pos	MG	3.233180547	0.044124903	1.52650149	up
MG(16:1)	pos	MG	4.165171084	0.002180809	2.321229006	up
MG(20:4)	pos	MG	1.985146418	0.02486743	1.263878334	up
MG(22:6)	pos	MG	2.153626007	0.016120367	1.314478324	up
Phosphatidic acid（17）						
PA(16:1)	pos	PA	12.46373157	0.013231923	1.769027822	up
PA(16:0/16:0)	neg	PA	1.587210937	0.036756267	1.116623496	up
PA(16:0/16:1)	neg	PA	2.974355124	0.027204936	1.355915949	up
PA(16:0/18:1)	neg	PA	1.927733874	0.043842769	1.165229953	up
PA(16:1/18:1)	neg	PA	7.81981789	1.05946E-05	1.45799447	up
PA(16:1/18:1)	neg	PA	3.029137778	0.000268711	1.212343994	up
PA(18:0/16:1)	neg	PA	2.982005663	0.00025543	1.459787983	up
PA(18:0/16:1)	neg	PA	1.671822782	0.013938753	1.054568675	up
PA(18:0/18:2)	neg	PA	1.930927767	0.013192865	2.075960745	up
PA(42:0)	neg	PA	1.80003251	0.028648894	1.016320471	up
PA(18:1/18:3)	neg	PA	0.452388958	0.010178356	1.352420617	down
PA(18:3/28:6)	neg	PA	0.336944249	0.009040028	1.636304951	down
PA(23:0/18:1)	neg	PA	0.418034584	0.001004882	1.93135236	down
PA(23:0/18:1)	neg	PA	0.676089078	0.036618031	1.035972672	down
PA(24:0/18:1)	neg	PA	0.63129021	0.004331539	1.126235106	down
PA(26:5/22:6)	neg	PA	0.672145145	0.036823671	1.430935197	down
PA(26:5/22:6)	neg	PA	0.509549127	0.005447778	2.456479625	down
Phosphatidylcholine（125）						
PC(14:0/15:2)	pos	PC	7.656217636	0.001049439	2.265068961	up
PC(14:0/15:2)	pos	PC	2.660875813	0.008062362	1.511169391	up
PC(15:0/19:4)	pos	PC	3.438828308	0.01532254	1.356681864	up
PC(15:2/22:6)	pos	PC	2.368732943	0.015421094	1.659988165	up

PC(16:0/18:2)	pos	PC	2.219758048	0.024645392	1.656541517	up
PC(16:1/18:1)	pos	PC	2.83444168	0.006547449	1.079055481	up
PC(16:1/19:2COOH)	pos	PC	3.378074412	0.003894471	1.316919374	up
PC(16:2/21:6)	pos	PC	2.608367808	0.044018828	1.481491818	up
PC(16:2/21:6)	pos	PC	1.948175385	0.030866852	1.246284226	up
PC(16:3/15:0)	pos	PC	2.299048633	0.013991297	1.576658629	up
PC(18:2/13:1)	pos	PC	6.222259786	0.00050832	1.69828186	up
PC(18:2/13:1)	pos	PC	2.481431884	0.008860374	1.087720537	up
PC(18:2/21:4COOH)	pos	PC	2.465086978	0.026564331	1.343594733	up
PC(18:4/15:2)	pos	PC	6.411442951	0.00027749	1.868934258	up
PC(18:4/15:2)	pos	PC	2.728170165	0.005077076	1.281774584	up
PC(18:4/18:1COOH)	pos	PC	2.577521139	0.012119487	2.027605256	up
PC(18:4/19:0COOH)	pos	PC	4.748579686	0.000902579	1.151093269	up
PC(18:4CHO)	pos	PC	2.410482286	0.049637637	1.416046082	up
PC(18:4COOH/18:0)	pos	PC	2.356223878	0.014995004	1.382472329	up
PC(20:5/14:0COOH)	pos	PC	3.702846257	0.032554647	2.387973417	up
PC(24:1/15:4COOH)	pos	PC	2.286035774	0.004917927	1.148645605	up
PC(31:2)	pos	PC	3.77385768	0.01411106	1.250818397	up
PC(32:1CHO)	pos	PC	4.482731382	0.001932427	1.873716863	up
PC(32:1CHO)	pos	PC	2.547402089	0.004903817	1.780156437	up
PC(32:2COOH)	pos	PC	5.07165031	0.000232056	1.089603078	up
PC(33:5)	pos	PC	3.731606505	0.001096807	2.388027203	up
PC(33:5)	pos	PC	2.458053091	0.015760835	1.64056497	up
PC(33:7)	pos	PC	4.115351254	0.002929874	1.330280347	up
PC(36:2COOH)	pos	PC	1.757646298	0.03923835	1.067237842	up
PC(36:6)	pos	PC	3.521630938	0.002287745	1.1444193	up
PC(40:4COOH)	pos	PC	3.165420177	0.001563257	1.646861826	up
PC(40:4COOH)	pos	PC	2.519661089	0.049788078	1.054210127	up
PC(46:8)	pos	PC	2.038282071	0.047309968	1.247243841	up

PC(14:1/20:1)	pos	PC	0.566717499	0.041487512	1.027739881	down
PC(15:0/18:0)	pos	PC	0.400169765	0.013185565	1.227347962	down
PC(15:0/20:5)	pos	PC	0.283184581	0.000883385	2.000748126	down
PC(17:0/18:2)	pos	PC	0.400043342	0.049357507	1.959603325	down
PC(18:1/17:0)	pos	PC	0.392909538	0.038163448	1.916330126	down
PC(18:3/15:0)	pos	PC	0.236056904	0.000146526	1.673980725	down
PC(18:3/15:0)	pos	PC	0.427207962	0.009605858	1.46006885	down
PC(18:3/16:0)	pos	PC	0.451656241	0.036811942	1.516183974	down
PC(19:4/18:0)	pos	PC	0.372674842	0.020562652	1.936269108	down
PC(22:2)	pos	PC	0.18914768	0.044999383	1.919766839	down
PC(22:5/18:0)	pos	PC	0.405860793	0.013776154	1.063115224	down
PC(28:3)	pos	PC	0.316808924	0.005320651	1.753403458	down
PC(28:3)	pos	PC	0.354918673	0.007026323	2.050816438	down
PC(29:3)	pos	PC	0.530745945	0.04950177	1.222442671	down
PC(29:3)	pos	PC	0.212716361	0.000441718	2.759496248	down
PC(30:3)	pos	PC	0.24836147	0.048915431	2.505686075	down
PC(30:3)	pos	PC	0.363215115	0.014120204	2.000442391	down
PC(30:4)	pos	PC	0.375255583	0.017472921	1.201913241	down
PC(30:4)	pos	PC	0.35271955	0.015327866	2.297585277	down
PC(31:1)	pos	PC	0.211903035	0.000711168	1.549695053	down
PC(32:5)	pos	PC	0.379301603	0.025605063	1.272120229	down
PC(32:5)	pos	PC	0.403116347	0.035594864	1.697652392	down
PC(33:3)	pos	PC	0.165348934	0.002128662	1.231375266	down
PC(35:0)	pos	PC	0.31247096	0.038247025	1.770123982	down
PC(36:2CHO)	pos	PC	0.441185626	0.016266367	1.152330319	down
PC(36:7COOH)	pos	PC	0.154127258	0.014210062	2.565076467	down
PC(37:2)	pos	PC	0.490110443	0.034825322	1.64522984	down
PC(38:2CHO)	pos	PC	0.287104657	0.001125465	2.833995489	down
PC(38:3CHO)	pos	PC	0.341801838	0.013108336	1.941201816	down

PC(38:4CHO)	pos	PC	0.346500341	0.009458974	1.837911856	down
PC(39:5)	pos	PC	0.402106108	0.022428763	1.814836977	down
PC(40:2CHO)	pos	PC	0.101777327	4.07212E-05	4.012159458	down
PC(40:2CHO)	pos	PC	0.365006211	0.003096879	2.595098829	down
PC(40:4CHO)	pos	PC	0.465032466	0.022103246	1.762077072	down
PC(41:2)	pos	PC	0.371726402	0.009425075	1.715726953	down
PC(42:3)	pos	PC	0.307280814	0.006554483	1.662915193	down
PC(42:3)	pos	PC	0.42825759	0.027289553	1.841816059	down
PC(15:2COOH/18:2)	neg	PC	2.562993016	0.003523344	1.510924807	up
PC(16:1/17:5CHO)	neg	PC	1.581268104	0.021221622	1.787831061	up
PC(17:0/16:1)	neg	PC	2.267728593	0.001404864	1.293152812	up
PC(17:0/16:1)	neg	PC	1.832944722	0.010032488	1.321378125	up
PC(19:4COOH/18:2)	neg	PC	1.754653085	0.028581291	2.012002761	up
PC(12:1/18:3)	neg	PC	0.323698959	0.003793747	1.403282398	down
PC(14:0/15:0)	neg	PC	0.123391818	0.001213797	1.867932512	down
PC(14:0/15:0)	neg	PC	0.178338471	0.012762376	2.147793247	down
PC(15:0/15:0)	neg	PC	0.240853792	0.00047849	2.635140901	down
PC(15:0/15:0)	neg	PC	0.369075111	0.006578743	2.350224597	down
PC(15:0/18:3)	neg	PC	0.155016485	0.000231953	1.825348433	down
PC(15:0/18:3)	neg	PC	0.325045889	0.015968992	1.220132848	down
PC(15:0/20:4)	neg	PC	0.146267803	7.80168E-05	3.46548539	down
PC(15:0/20:4)	neg	PC	0.334059356	0.000531832	2.27505382	down
PC(15:0/22:5)	neg	PC	0.421961515	0.010136831	2.467628814	down
PC(15:0/22:5)	neg	PC	0.426374305	0.014540018	1.774598786	down
PC(15:1/18:3)	neg	PC	0.248574094	0.000363775	1.604339264	down
PC(15:1/20:4)	neg	PC	0.500783386	0.012972504	1.987290695	down
PC(16:0/19:1COOH)	neg	PC	0.302732783	0.000194413	1.54654351	down
PC(16:0/19:4)	neg	PC	0.778660752	0.048101584	1.275052822	down
PC(16:0/19:4)	neg	PC	0.356435705	0.008322995	1.122231112	down

PC(16:0/22:5)	neg	PC	0.447500297	0.020357912	1.106081933	down
PC(16:1/16:0)	neg	PC	0.406418696	0.019751468	1.842310042	down
PC(16:1/19:1)	neg	PC	0.440881424	0.007240078	1.828051238	down
PC(16:1/19:1)	neg	PC	0.480784728	0.002884146	1.513874058	down
PC(16:1/22:5)	neg	PC	0.468347194	0.008410266	1.395625732	down
PC(16:2/18:3)	neg	PC	0.405292101	0.008839088	1.657547648	down
PC(16:2/22:4)	neg	PC	0.566179029	0.039154183	1.090683418	down
PC(18:0/17:1)	neg	PC	0.187750942	0.000240245	2.40748317	down
PC(18:0/17:1)	neg	PC	0.414606357	0.002670393	1.417459051	down
PC(18:0/20:1)	neg	PC	0.475662051	0.010803225	1.616312054	down
PC(18:0/22:4)	neg	PC	0.569328065	0.047913068	1.316232408	down
PC(18:0/22:5)	neg	PC	0.414332476	0.011953442	1.323167452	down
PC(18:1/19:2)	neg	PC	0.558477853	0.008137541	1.007630187	down
PC(18:2/18:2)	neg	PC	0.475491242	0.049511393	1.390537646	down
PC(18:2/19:2)	neg	PC	0.51931336	0.016571162	1.445013405	down
PC(18:2/22:6)	neg	PC	0.383213297	0.008914297	1.52157305	down
PC(18:4/18:1)	neg	PC	0.470350763	0.002166312	1.308048962	down
PC(18:4COOH/18:1)	neg	PC	0.38776054	0.011025403	1.870340386	down
PC(18:4COOH/18:1)	neg	PC	0.46192647	0.035118116	1.834994983	down
PC(19:0/18:1)	neg	PC	0.38515281	0.000949302	1.564214243	down
PC(19:0/18:2)	neg	PC	0.362098138	0.001307346	2.129683639	down
PC(19:0/18:2)	neg	PC	0.550192393	0.037866221	1.509631823	down
PC(19:4COOH/24:0)	neg	PC	0.410810879	0.00962223	1.091018059	down
PC(20:0/18:2)	neg	PC	0.551007058	0.022684074	1.027725256	down
PC(20:3COOH/18:2)	neg	PC	0.405597789	0.001223369	1.865645047	down
PC(20:3COOH/18:2)	neg	PC	0.573129824	0.030573694	1.558121086	down
PC(21:4/21:4COOH)	neg	PC	0.236517746	0.011181799	1.848158023	down
PC(23:0/18:1)	neg	PC	0.603955869	0.011026365	2.240596941	down
PC(23:0/18:1)	neg	PC	0.267400909	4.15665E-05	2.429609303	down

PC(23:0/18:2)	neg	PC	0.211659808	0.001811756	2.584569592	down
PC(23:0/18:2)	neg	PC	0.34776808	0.004155047	2.064731792	down
PC(24:0/18:2)	neg	PC	0.55081334	0.021849278	2.092981497	down
PC(24:0/18:2)	neg	PC	0.330651678	0.003581257	2.252329473	down
PC(37:4COOH)	neg	PC	0.397780104	0.007925816	1.425335453	down
Phosphatidyl ethanolamine （83）						
PE(14:0/16:1)	pos	PE	2.809962573	0.030710293	1.027104362	up
PE(14:0/18:2)	pos	PE	9.15655524	0.000518473	2.201499337	up
PE(14:0/18:2)	pos	PE	2.556538404	0.013026875	1.278874255	up
PE(14:1/20:2)	pos	PE	3.900256238	0.000434311	1.709136839	up
PE(16:0/18:2)	pos	PE	4.234071927	0.000472923	2.629668347	up
PE(16:0/18:2)	pos	PE	2.267838876	0.020042489	1.538293727	up
PE(16:0/18:2CHO)	pos	PE	10.2803324	0.000211632	1.90779182	up
PE(16:0/18:2CHO)	pos	PE	3.147980857	0.006114062	1.335068248	up
PE(16:0/20:4)	pos	PE	2.702406485	0.009753861	1.677867108	up
PE(16:1/18:1)	pos	PE	4.475013628	0.003745482	1.542542698	up
PE(18:1/16:1)	pos	PE	4.475013628	0.003745482	1.58834241	up
PE(18:1/18:2)	pos	PE	2.588443408	0.01527473	1.848328976	up
PE(18:3/16:1)	pos	PE	2.996980625	0.003235554	1.302175777	up
PE(18:4/16:1)	pos	PE	2.959244516	0.003980226	1.268264286	up
PE(20:5/16:1)	pos	PE	8.407976083	0.000154657	1.716452141	up
PE(20:5/16:1)	pos	PE	2.646540858	0.00744725	1.033783231	up
PE(34:2CHO)	pos	PE	2.541070913	0.003123778	1.097035014	up
PE(36:4)	pos	PE	2.674810158	0.021103047	1.440169801	up
PE(36:5)	pos	PE	4.742646912	0.000364025	2.705273757	up
PE(36:5)	pos	PE	2.34906439	0.022002954	1.497725863	up
PE(36:7)	pos	PE	5.181048341	0.012224676	1.506285618	up
PE(38:6)	pos	PE	3.652644051	0.003053086	1.934453218	up
PE(18:0/22:2CHO)	pos	PE	0.304115769	0.001827291	1.218133085	down

PE(18:0/22:2CHO)	pos	PE	0.472476919	0.026234801	1.222387593	down
PE(18:0/22:4)	pos	PE	0.449596356	0.033659649	1.495792439	down
PE(18:1/22:2CHO)	pos	PE	0.188822323	0.001415544	3.242589378	down
PE(18:1/22:2CHO)	pos	PE	0.371634716	0.041993306	2.864145057	down
PE(22:3CHO/18:1)	pos	PE	0.325039143	0.034278797	1.533670038	down
PE(40:3CHO)	pos	PE	0.362467231	0.043829129	1.928096382	down
PE(16:1/18:2)	neg	PE	4.001778621	0.000443237	1.670938147	up
PE(16:1/18:2)	neg	PE	1.900686952	0.026943914	1.220852304	up
PE(16:1/19:3COOH)	neg	PE	3.573731416	0.001278007	1.970063628	up
PE(16:1/20:4)	neg	PE	2.489554429	0.008638088	1.543233088	up
PE(16:1/20:5)	neg	PE	3.505650783	0.034281194	3.149632133	up
PE(17:1CHO/18:2)	neg	PE	1.694569474	0.048191821	1.017836972	up
PE(18:0/18:2)	neg	PE	2.928234989	0.033304916	2.062438818	up
PE(18:2/18:2)	neg	PE	1.893782821	0.025583219	1.772592263	up
PE(18:2/19:3COOH)	neg	PE	3.083122473	0.001520178	1.80934254	up
PE(18:2/21:4COOH)	neg	PE	2.416539493	0.011014834	2.639408479	up
PE(19:2COOH/20:5)	neg	PE	1.70235245	0.025963871	1.34572256	up
PE(12:0/17:0)	neg	PE	0.373759656	0.0014931	1.477777175	down
PE(14:0/15:0)	neg	PE	0.185863101	0.007629306	3.846424442	down
PE(14:0/22:2CHO)	neg	PE	0.269320373	0.004400595	1.535842901	down
PE(14:0/22:2CHO)	neg	PE	0.255345728	0.003543798	1.768736857	down
PE(14:1/16:0)	neg	PE	0.257458695	0.009102519	1.956344256	down
PE(14:1/16:0)	neg	PE	0.304754373	0.034773388	1.889711481	down
PE(15:0/16:0)	neg	PE	0.265510546	0.00271321	1.321939141	down
PE(15:0/16:0)	neg	PE	0.417669402	0.038055611	1.062038616	down
PE(15:0/18:3)	neg	PE	0.273955579	0.00203535	1.685486063	down
PE(15:0/18:3)	neg	PE	0.339397246	0.007791381	1.824787071	down
PE(15:1/16:0)	neg	PE	0.366421976	0.005191321	2.074287598	down
PE(15:1/16:0)	neg	PE	0.330159325	0.004969358	1.837792268	down

PE(15:1/18:2)	neg	PE	0.594152244	0.026943622	1.075709053	down
PE(15:1/18:3)	neg	PE	0.307965854	0.000786112	1.788678471	down
PE(15:1/18:3)	neg	PE	0.441657733	0.007517768	1.352300224	down
PE(15:1/20:4)	neg	PE	0.478754212	0.010872767	1.157701124	down
PE(16:1/15:0)	neg	PE	0.297335601	0.020438049	1.794055973	down
PE(16:1/15:0)	neg	PE	0.17422387	0.023808701	2.829890601	down
PE(16:1/17:0)	neg	PE	0.340100105	0.004837832	1.901700896	down
PE(16:1/17:0)	neg	PE	0.339000323	0.002676593	1.652429789	down
PE(17:0/18:0)	neg	PE	0.285956065	0.001700148	2.431121421	down
PE(17:0/18:0)	neg	PE	0.246384055	0.001060323	2.885878907	down
PE(17:0/18:1)	neg	PE	0.368576921	0.002839632	1.561489235	down
PE(17:0/22:2CHO)	neg	PE	0.104184892	2.33717E-05	2.671655397	down
PE(17:0/22:2CHO)	neg	PE	0.232559351	0.00025038	1.90347654	down
PE(17:1/22:3)	neg	PE	0.423821521	0.024760528	1.826982191	down
PE(17:1/22:5)	neg	PE	0.219294588	3.34359E-05	2.473554624	down
PE(17:1/22:5)	neg	PE	0.512099116	0.018052225	1.386745772	down
PE(18:0/18:0)	neg	PE	0.306625607	0.049387301	1.844357534	down
PE(18:0/20:3)	neg	PE	0.465091407	0.01957033	1.206125822	down
PE(18:0/22:1)	neg	PE	0.671753082	0.037442965	1.692388188	down
PE(18:0/22:2COOH)	neg	PE	0.291560081	0.013281477	1.056031501	down
PE(18:0/22:2COOH)	neg	PE	0.297297053	0.007024269	1.176935499	down
PE(18:0/22:3)	neg	PE	0.496084316	0.03057902	1.0449858	down
PE(18:1/17:0)	neg	PE	0.316038708	0.00235485	2.608206736	down
PE(18:1/18:1)	neg	PE	0.520426616	0.027932306	1.467107536	down
PE(20:0/18:3)	neg	PE	0.446240738	0.034026881	2.006661189	down
PE(20:2COOH/18:1)	neg	PE	0.384903742	0.015590313	1.326206247	down
PE(22:0/18:1)	neg	PE	0.676325986	0.035871033	1.617857042	down
PE(23:0/21:3COOH)	neg	PE	0.528281577	0.02924984	1.336484371	down
PE(23:0/21:3COOH)	neg	PE	0.480433599	0.019041778	1.451036661	down
PE(24:0/18:1)	neg	PE	0.382056657	0.019322764	1.92014498	down

PE(9:0CHO/18:0)	neg	PE	0.383966572	0.000192512	2.400160129	down
Phosphatidylethanolamine ethyl ester（2）						
PEt(18:4/21:2)	pos	PEt	4.063027387	0.001315684	1.62569474	up
PEt(20:0)	pos	PEt	3.130726416	0.022807025	1.04031163	up
Perfluoroalkyl acid（1）						
PFAA(15:0)	pos	PFAA	2.155224487	0.039800869	1.386475604	up
Phosphatidylinositol（11）						
PI(12:0/18:0)	neg	PI	2.469748843	0.003740443	2.130315343	up
PI(12:0/18:0)	neg	PI	1.753268	0.030888663	2.001450153	up
PI(16:0/18:1)	pos	PI	2.253198231	0.0158434	1.409257813	up
PI(16:0/20:3)	pos	PI	3.318718654	0.00525211	1.57898552	up
PI(16:0/20:3)	pos	PI	2.316761709	0.022038464	1.133062278	up
PI(18:0/16:1)	pos	PI	2.322130827	0.011447235	1.590940117	up
PI(18:0/22:5)	neg	PI	0.496455374	0.035935497	1.109829211	down
PI(18:1/22:5)	neg	PI	0.22787565	0.03204178	1.843197301	down
PI(19:0/20:4)	neg	PI	0.286412637	0.000850572	2.808417868	down
PI(22:7/16:0)	pos	PI	2.861599772	0.00066495	1.766691064	up
PI(22:7/16:0)	pos	PI	2.063221547	0.017147884	1.223606056	up
Phosphatidylserine（27）						
PS(18:3/18:0)	pos	PS	6.497425338	0.000395078	1.78459442	up
PS(18:3/18:0)	pos	PS	2.518510764	0.014369371	1.370361301	up
PS(19:0/18:2)	neg	PS	2.26051757	0.017534902	2.079098012	up
PS(19:0/18:2)	neg	PS	2.316580191	0.016865675	2.900444064	up
PS(22:1/18:2)	neg	PS	1.883771308	0.006073732	1.214451727	up
PS(22:1/18:2)	neg	PS	1.557307872	0.045705776	1.165534485	up
PS(22:1/20:4)	neg	PS	2.170696109	0.000396797	1.312860874	up
PS(22:1/20:4)	neg	PS	2.220759003	0.002972801	1.733674656	up
PS(15:0/20:1)	neg	PS	0.531786082	0.003480815	1.284448532	down
PS(17:0/18:1)	neg	PS	0.432372202	0.00171963	1.103413395	down

PS(18:0/19:2)	neg	PS	0.04793149	0.007348867	2.592972388	down
PS(18:0/19:2)	neg	PS	0.044848272	0.005934744	3.197594267	down
PS(18:0/20:2)	neg	PS	0.59285544	0.040994335	1.220299008	down
PS(18:1/23:1)	neg	PS	0.436332329	0.000985824	1.089748573	down
PS(18:1/25:1)	neg	PS	0.436789973	0.010877393	1.06523262	down
PS(19:0/22:4)	neg	PS	0.35466424	0.00021315	2.445404898	down
PS(20:0/22:4)	neg	PS	0.594511361	0.010376319	1.406747538	down
PS(21:0/20:3)	neg	PS	0.522080214	0.007824803	1.308234877	down
PS(22:0/22:5)	neg	PS	0.437147783	0.001465944	1.433815646	down
PS(23:0/18:1)	neg	PS	0.351963245	0.000308746	1.276083808	down
PS(23:0/18:2)	neg	PS	0.479901557	0.021972912	1.213367752	down
PS(23:0/18:3)	neg	PS	0.232944263	5.11618E-05	1.785549658	down
PS(23:0/18:3)	neg	PS	0.51486534	0.024287256	1.029012201	down
PS(23:0/20:4)	neg	PS	0.34921585	0.001185018	1.982369065	down
PS(23:0/20:5)	neg	PS	0.413010723	0.021061787	1.200564246	down
PS(25:0/18:1)	neg	PS	0.370662669	0.002732694	1.564614144	down
PS(25:0/18:2)	neg	PS	0.523964403	0.015788354	1.361874293	down
Sphingomyelin（16）						
SM(d18:2/20:1)	pos	SM	3.434533038	0.007217946	1.579038366	up
SM(d18:2/20:1)	pos	SM	1.808038327	0.048877368	1.227109671	up
SM(d28:1)	pos	SM	3.954373022	0.00016752	1.27062443	up
SM(d28:2)	pos	SM	3.898133919	0.000143295	1.466102548	up
SM(d32:3)	pos	SM	3.359990449	0.037221603	1.286097493	up
SM(d34:0)	pos	SM	2.840513561	0.027263798	1.59122228	up
SM(d34:1)	pos	SM	2.50000968	0.0119731	1.670948983	up
SM(d36:0)	pos	SM	3.203627045	0.006440693	1.0983343	up
SM(d36:1)	pos	SM	3.199225848	0.021044604	1.649700088	up
SM(d38:1)	pos	SM	4.68524095	0.010276851	1.898536617	up
SM(d38:5)	pos	SM	2.622540298	0.027822663	1.367049654	up
SM(d39:6)	pos	SM	2.122407366	0.03840126	1.538076841	up

SM(t32:0)	pos	SM	2.561315152	0.023914722	1.291818939	up
SM(t34:0)	pos	SM	2.746310935	0.009353319	1.911937503	up
SM(t36:1)	pos	SM	2.807143472	0.018414441	1.106650089	up
SM(d33:4)	pos	SM	0.425758248	0.02364502	1.924308651	down
Sphingosine（3）						
SPH(d16:1)	pos	SPH	3.245571304	0.006194247	1.995350824	up
SPH(d18:1)	pos	SPH	4.021276555	0.000125048	2.844136924	up
SPH(d18:1)	pos	SPH	2.59447201	0.002279977	2.106797494	up
Sphingosine-1-phosphate（2）						
SPHP(d18:1)	neg	SPHP	4.551653665	0.00411672	2.867821366	up
SPHP(d18:1)	neg	SPHP	2.33620408	0.014848326	2.842659553	up
Triacylglycerol（10）						
TG(14:0/14:0/18:0CHO)	pos	TG	5.890527721	0.045267795	1.518735484	up
TG(14:0/15:0/18:1)	pos	TG	2.134816999	0.021578784	1.399526643	up
TG(14:0/18:0/18:1CHO)	pos	TG	2.323113736	0.031849869	1.291716241	up
TG(14:1/16:1/18:1)	pos	TG	2.148652137	0.014772499	1.183342353	up
TG(15:0/19:0CHO/18:1)	pos	TG	5.277282888	0.046035699	1.585465832	up
TG(19:0CHO/13:0/16:1)	pos	TG	3.842199818	0.023186976	1.198634033	up
TG(2:0/4:0/18:1)	pos	TG	22.92684255	0.031708868	1.172414587	up
TG(32:0/19:2COOH)	pos	TG	5.233725165	0.038701949	1.188983892	up
TG(34:0CHO/16:1)	pos	TG	5.277050521	0.045103941	1.296807417	up
TG(4:1CHO/18:1/18:1)	pos	TG	8.807411978	0.046397708	1.496000477	up
Wax ester（6）						
WE(18:1)	pos	WE	2.262149263	0.00742617	1.467980893	up
WE(24:0/18:3)	pos	WE	3.040089785	0.013442016	1.872596268	up
WE(34:3)	pos	WE	2.127240152	0.048783321	1.359145562	up
WE(42:5)	pos	WE	3.808787163	0.002386383	1.49643955	up
WE(42:5)	pos	WE	2.484112897	0.007969847	1.224213847	up
WE(43:4)	pos	WE	2.018958973	0.025581415	1.078274776	up
